# Dynamics of Tri-Hybrid Nanoparticles in the Rheology of Pseudo-Plastic Liquid with Dufour and Soret Effects

**DOI:** 10.3390/mi13020201

**Published:** 2022-01-27

**Authors:** Enran Hou, Fuzhang Wang, Umar Nazir, Muhammad Sohail, Noman Jabbar, Phatiphat Thounthong

**Affiliations:** 1 College of Mathematics, Huaibei Normal University, Huaibei 235000, China; houenran@163.com; 2 Department of Mathematics, Nanchang Institute of Technology, Nanchang 330044, China; wangfuzhang1984@163.com; 3 College of Mathematics and Statistics, Xuzhou University of Technology, Xuzhou 221018, China; 4 Department of Applied Mathematics and Statistics, Institute of Space Technology, P.O. Box 2750, Islamabad 44000, Pakistan; nazir_u2563@yahoo.com (U.N.); noman.jabbar777@gmail.com (N.J.); 5 Renewable Energy Research Centre, Department of Teacher Training in Electrical Engineering, Faculty of Technical Education, King Mongkut’s University of Technology North Bangkok, 1518 Pracharat 1 Road, Bangsue, Bangkok 10800, Thailand; phatiphat.t@fte.kmutnb.ac.th

**Keywords:** tri-hybrid nanoparticles, Soret and Dufour effect, boundary layer analysis, finite element scheme, heat generation, constructive and destructive chemical reaction

## Abstract

The rheology of different materials at the micro and macro levels is an area of great interest to many researchers, due to its important physical significance. Past experimental studies have proved the efficiency of the utilization of nanoparticles in different mechanisms for the purpose of boosting the heat transportation rate. The purpose of this study is to investigate heat and mass transport in a pseudo-plastic model past over a stretched porous surface in the presence of the Soret and Dufour effects. The involvement of tri-hybrid nanoparticles was incorporated into the pseudo-plastic model to enhance the heat transfer rate, and the transport problem of thermal energy and solute mechanisms was modelled considering the heat generation/absorption and the chemical reaction. Furthermore, traditional Fourier and Fick’s laws were engaged in the thermal and solute transportation. The physical model was developed upon Cartesian coordinates, and boundary layer theory was utilized in the simplification of the modelled problem, which appears in the form of coupled partial differential equations systems (PDEs). The modelled PDEs were transformed into corresponding ordinary differential equations systems (ODEs) by engaging the appropriate similarity transformation, and the converted ODEs were solved numerically via a Finite Element Procedure (FEP). The obtained solution was plotted against numerous emerging parameters. In addition, a grid independent survey is presented. We recorded that the temperature of the tri-hybrid nanoparticles was significantly higher than the fluid temperature. Augmenting the values of the Dufour number had a similar comportment on the fluid temperature and concentration. The fluid temperature increased against a higher estimation of the heat generation parameter and the Eckert numbers. The impacts of the buoyancy force parameter and the porosity parameter were quite opposite on the fluid velocity.

## 1. Introduction

In the last few decades, many researchers have become interested in the study of shear thinning fluids, due to the many fascinating industrial and everyday applications [1]. A small number of these include wall paint, printing ink, nail polish, whipped cream, ketchup, and engine oil. Shear thinning fluid can also be called pseudo-plastic fluid and is considered to display the behaviors of both Newtonian fluid and of plastic fluid. In shear thinning fluid, the more stress is applied, the more freely the fluid flows. This property is a useful characteristic for its use in materials such as paint, oils, and cream. Eberhard et al. [2] computed the effective viscosity for Newtonian and non-Newtonian materials past through a porous surface. They first considered the rheology of the power law model to estimate an effective shear rate. In their investigation, they assumed the constant permeability. Rosti and Takagi [3] studied the shear thinning and shear thickening behaviors of materials by studying different important aspects. Moreover, they recorded several important features through changing the phase and volume fractions. Gul et al. [4] computed the exact solution in the case of lifting and drainage with slip conditions for the power law model of thin film. They approximated the flow rate and the skin friction coefficient and plotted numerous sketches against different involved parameters for fluid velocity. They recorded the decline in the velocity field for the escalating values of the slip parameter. Pseudo-plastic nanofluid obeying Brownian motion and thermophoresis past over a vertical cylinder was examined by Hussein et al. [5]. They solved the converted modelled equations numerically and monitored the decline in velocity against the curvature parameter and fluid parameter. Abdelsalam and Sohail [6] studied the involvement of the bio-convection phenomenon in viscous nanofluid comprising variable properties past over a bidirectional stretched surface engaging an optimal homotopy scheme. They established an error analysis and performed a comparative study, noting the depreciation in the motile density profile against the Peclet and Lewis numbers. Sohail and Naz [7] investigated the stretched Sutterby fluid flow in a cylinder and presented the dynamical survey while considering thermophoresis, Brownian motion; thermal and concentration relaxation times. They used the polar coordinates to derive the physical model in the form of coupled partial differential equations systems (PDEs) and then transformed these into ordinary differential equations systems (ODEs) by engaging the appropriate similarity transformation while utilizing the approach of boundary layer theory. Afterwards, converted ODEs were tackled analytically. They monitored the decline in fluid temperature against the Prandtl number and the concentration was controlled for the higher values of the Schmidt number. Chu et al. [8] examined the involvement of chemical reaction and activation energy in the nanofluid flow problem. They solved the resulting equations numerically via a finite element procedure, recording the decline in the fluid velocity against the magnetic parameter. Hina et al. [9] used a long wavelength approach to model the pseudo-plastic fluid problem with wall and slip properties in a curved channel under the peristaltic transport phenomenon. They solved the boundary value problem numerically and the solution was plotted for different parametric values in Mathematica 15.0 software; they also examined the symmetric pattern for fluid velocity against a larger curvature parameter. Salahuddin et al. [10] numerically stimulated the magnetohydrodynamic (MHD) flow for a pseudo-plastic fluid over a stretching cylinder with a MHD effect and temperature dependent thermal conductivity. They derived the physical model into a mathematical form by engaging boundary layer theory. The derived problem was highly nonlinear in nature and was presented in the form of PDEs. Similarity transformation was used to simplify the problem and to convert PDEs into ODEs. A numerical solution was obtained via the Keller box scheme, and they recorded the decline in fluid velocity for higher values of the Weissenberg number. Alam et al. [11] presented a study on the drainage and lifting MHD pseudo-plastic problem and addressed the exact solution, finding the decline in velocity against the Stokes number. The phenomenon of tapering is observed in the peristaltic flow of the pseudo-plastic model with variable viscosity studied by Hayat et al. [12]. They engaged a perturbation approach to solving the boundary value problem in the symmetric channel and observed an increase in velocity against the higher values of the magnetic parameter. Moreover, they plotted streamlines for different parameters. Further important contributions are reported in [13,14,15].

The principle behind synthesizing nanofluid composites is to enhance the properties of a single nanoparticle that possesses either improved thermal conductivity or improved rheological properties. By framing, nanofluids are made using single nanoparticles which are useful in developing a greater ability to absorb heat energy and the rheological properties are improved in various fluids. In a similar way, a composite of nanofluids is most significant in view of an improved transfer of thermal energy and rheological properties in liquids. Composites of nanoparticles are known as nanofluids, while composites of two or more nanofluids are called hybrid nanoparticles. Composites of three kinds of nanofluids are known as tri-hybrid nanoparticles. Tri-hybrid nanoparticles are recognized as the most significant to the betterment of thermal conductivity. Applications of such composite particles are relevant in the making of electronic heaters, the production of solar energy, nuclear safety, the pharmaceutical industry, etc. 

Tri-hybrid nanoparticles have been studied by various scholars. For example, Manjunatha et al. [16] discussed the effect of tri-hybrid nanoparticles in energy transfer phenomena considering convective conditions. Nazir et al. [17] studied significant thermal growth for ternary hybrid nanoparticles as compared to the thermal growth for hybrid nanofluid and nanoparticles in complex fluid over a heated two-dimensional frame. They implemented a finite element approach to achieve numerical results. Chen et al. [18] scrutinized the thermal properties along with the Ternary hybrid nanostructures in graphene oxide/graphene and ${\mathrm{MoS}}_{2}$/zirconia while they found an improvement in the tribiological and mechanical properties. Zayan, Mohammed et al. [19] investigated novel ternary hybrid nanostructures in view of the thermal additives. Shafiq et al. [20] studied Walters’ B liquid in nanoparticles in view of dual stratification including the stagnation point using a Riga plate. Swain et al. [21] discussed features related to hybrid nanoparticles in the presence of a chemical reaction towards a stretching surface including slip conditions. Mebarek-Oudina et al. [22] performed a useful model study regarding hybrid nanoparticles under magnetic parameter in view of convection heat energy in a trapezoidal cavity. Warke et al. [23] numerically investigated the stagnation point behavior in the presence of thermal radiation impact over a heated surface. Dadheech et al. [24] captured the impacts related to heat energy transfer in the presence of hybrid nanoparticles using the role of hybrid nanoparticles under a magnetic parameter. Marzougui et al. [25] studied entropy generation and heat transfer including nanoparticles in a lid-driven cavity under a magnetic field. Oudina [26] simulated convective heat transfer using a category of nanoparticles based on titanium nanofluids using heat source terms. Dhif et al. [27] analyzed the role of hybrid nanofluid in solar collectors. Zamzari et al. [28] discussed the influences of mixed convection in a vertical heated channel whereby they determined aspects of entropy generation. Li et al. [29] determined the flow and the thermal characterizations in non-Newtonian liquid, adding nanoparticles using a non-Fourier approach alongside a Prandtl approach in the presence of a Darcy–Forchheimer motion. Mehrez et al. [30] investigated the impact of a magnetic field in ferro-fluid in a heated channel. Khashi’ie et al. [31] discussed the influences of hybrid nanofluid along with the shape factor while considering thermal radiation effect. Esfe et al. [32] analyzed the role of non-Newtonian liquid, adding hybrid nanoparticles to non-Newtonian material using variable viscosity. Mehrez and Cafsi [33] captured the role of hybrid nanofluid in a heated cavity via a pulsating inlet condition.

A surveying of the available literature shows that no adequate study has been performed involving ternary hybrid nanoparticles. This contribution aims to fill this gap in the research. A literature survey is covered in Section 1; the modeling is included in Section 2 with attention given to several important physical effects; the computational strategy is explained in Section 3; the results are analyzed in Section 4 and Section 5.

**Figure 1:**
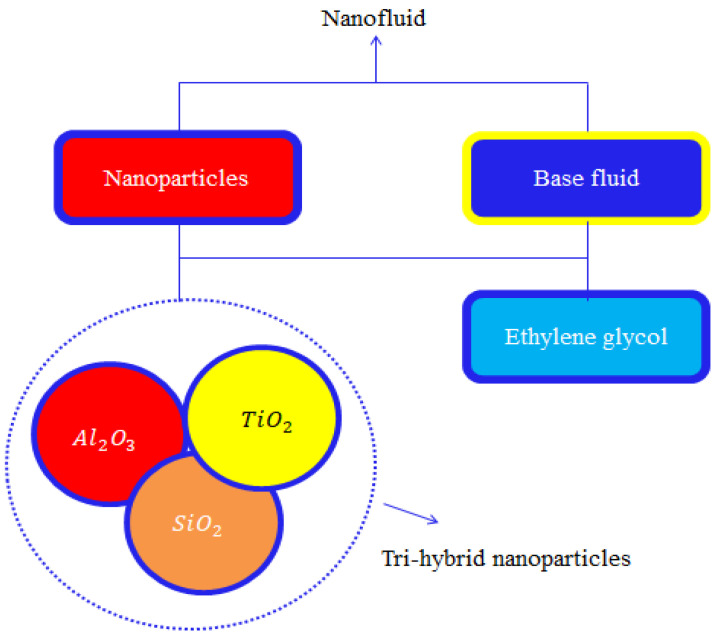
A description of the tri-hybrid approach in nanofluids.

The preparation approach associated with ternary hybrid nanoparticles is captured in Figure 1.

## 2. Description of Constructing Model

The rheology of a two-dimensional heat and mass diffusion transfer model in a pseudo-plastic liquid past a vertical surface was considered in the presence of a Darcy–Forchheimer model. A phenomenon associated with Dufour and Soret impacts was analyzed. The base fluid is assumed as ethylene glycol in a pseudo-plastic liquid inserting three kinds of nanoparticles (${\mathrm{Al}}_{2}O_{3},{\mathrm{SiO}}_{2}$, and ${\mathrm{TiO}}_{2}$). The thermal properties of silicon dioxide, ethylene glycol, aluminum oxide, and tritium dioxide are considered in Table 1. The following assumptions are detailed below.

**Figure 2:**
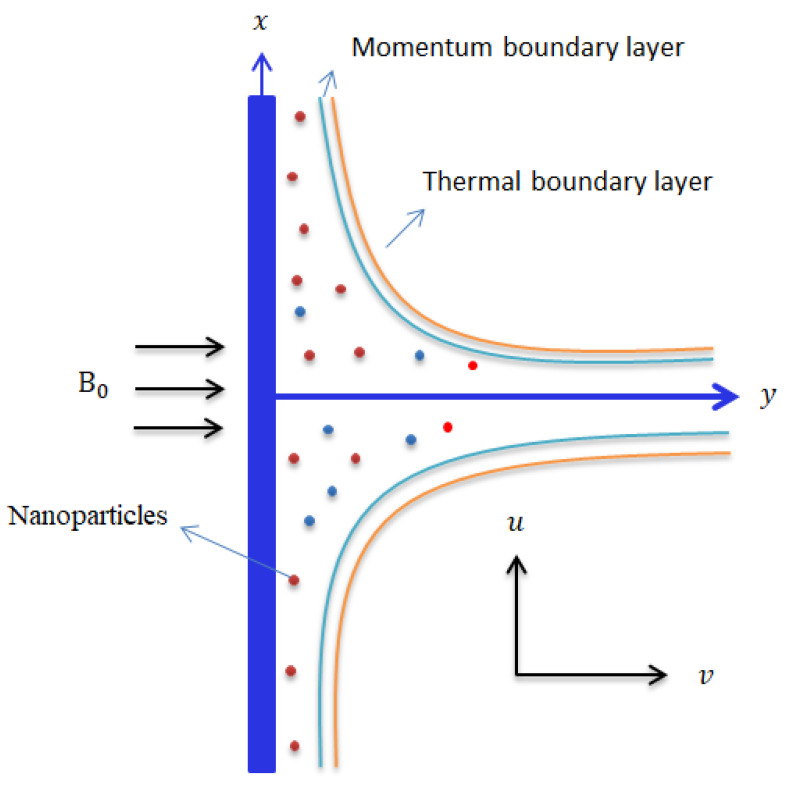
Illustration of the geometry of the current analysis.

- Two-dimensional flow of the pseudo-plastic material is considered;
- Fourier’s law and Fick’s law are assumed;
- Chemical reaction and heat generation are addressed;
- Transfer of heat is characterized in the presence of the Dufour and Soret effects;
- Viscous dissipation is removed;
- Darcy–Forchheimer porous theory is analyzed, and the vertical surface is plotted in Figure 2.

The power law model associated with shear stress is defined as:

(1) $$\tau_{xy}=-n\left({\left|\frac{\partial u}{\partial y}\right|}^{m-1}\right)\frac{\partial u}{\partial y}.$$

Equation (1) is known as the power law model regarding the shear stress of pseudo-plastic liquid. The fluids category is based on the numerical values of m. The present model becomes a Newtonian fluid model when m=1 while the present model can be converted into a dilatant fluid mode when m>1, and the present model becomes a pseudo-plastic liquid model when 0<m<1.

Boundary layer approximations are used to derive a system of PDEs on conservations laws regarding momentum, thermal energy, and mass diffusion. The present model is considered in terms of two-dimensional flow as well as steady and incompressible flow. The modeled PDEs (partial differential equations) are deduced Refs. [35,36,37] as:

(2) $$\frac{\partial u}{\partial x}+\frac{\partial v}{\partial y}=0,$$

(3) $$u\frac{\partial u}{\partial x}+v\frac{\partial v}{\partial y}=\nu_{tehnf}\frac{\partial }{\partial x}\left({\left|\frac{\partial u}{\partial y}\right|}^{m-1}\frac{\partial u}{\partial y}\right)-\frac{\nu_{tehnf}}{k^{s}}F_{D}u-\frac{F_{D}}{{\left(k^{s}\right)}^{\frac{1}{2}}}u^{2}+g\alpha \left(T-T_{\infty }\right)+g\beta \left(C-C_{\infty }\right),$$

(4) $$u\frac{\partial T}{\partial x}+v\frac{\partial T}{\partial y}=\frac{K_{tehnf}}{{\left(\rho C_{P}\right)}_{tehnf}}\frac{\partial^{2}T}{\partial y^{2}}+\frac{Q\left(T-T_{\infty }\right)}{{\left(\rho C_{P}\right)}_{tehnf}}+\frac{k_{t}D_{tehnf}}{{\left(C_{P}\right)}_{f}C_{s}}\frac{\partial^{2}C}{\partial^{2}y}+\frac{\mu_{tehnf}}{{\left(\rho C_{P}\right)}_{tehnf}}{\left|\frac{\partial u}{\partial y}\right|}^{m+1},$$

(5) $$u\frac{\partial C}{\partial x}+v\frac{\partial C}{\partial y}=D_{tehnf}\frac{\partial^{2}C}{\partial y^{2}}+\frac{k_{t}D_{tehnf}}{T_{M}}\frac{\partial^{2}T}{\partial y^{2}}-K\left(C-C_{\infty }\right).$$

Equation (2) is a continuity equation for two-dimensional flows as well as steady and incompressible flows. Equation (3) is termed as a momentum equation in the presence of pseudo-plastic liquid inserting correlations of tri-hybrid nanoparticles using bouncy forces, while Equations (4) and (5) are concentration and thermal energy equations including the effects of the chemical reaction; viscous dissipation; heat source; the Dufour and Soret influences. In Equation (3), the terms on the left-hand side are known as the inertial force, the first term on the right-hand side is the viscous force in the presence of pseudo-plastic liquid, whereas the last two terms on the right-hand side of Equation (3) are due bouncy forces and the two middle terms on the right-hand side of Equation (3) are modeled on Darcy–Forchheimer law. Terms on the left-hand side of Equation (4) are convection terms in heat transfer phenomena, the first term on the right-hand side is a conduction term in heat transfer phenomena, the second term on the right-hand side of Equation (4) occurs due to the heat source, while the third and last terms on the right-hand side of Equation (4) are formulated based on the effects of Soret and viscous dissipation. The second term on the right-hand side of Equation (5) is the Dufour effect, the last term on right-hand side signifies a chemical reaction, the first term on the right-hand side and the terms on the left-hand side occur due to the diffusion of mass species in view of convection and of conduction, respectively.

The desired (boundary conditions) BCs are:

(6) $$u=u_{w},\;v=-v_{w},\;C=C_{w},\;T=T_{w}\;\mathrm{at}\;y=0,\;u\to u_{\infty },\;C\to C_{\infty },\;T\to T_{\infty }\;\mathrm{when}\;y\to \infty .$$

Transformations are defined as:

(7) $$\theta =\frac{T-T_{\infty }}{T_{w}-T_{\infty }},\;\theta =\frac{C-C_{\infty }}{C_{w}-C_{\infty }},\;\xi =y{\left(\frac{U^{2-m}}{x\nu_{f}}\right)}^{\frac{1}{m+1}},\;\Psi =F{\left(x\nu_{f}U^{2m-1}\right)}^{\frac{1}{m+1}}.$$

Dimensionless ODEs are formulated via defined transformations:

(8) $${\left({\left|F^{\prime \prime }\right|}^{m-1}F^{\prime \prime }\right)}^{\prime }+\frac{1}{m+1}F^{\prime \prime }F-\epsilon F^{\prime }-\frac{\nu_{f}}{\nu_{tehnf}}F_{R}\left({F^{\prime }}^{2}\right)+\frac{\nu_{f}}{\nu_{tehnf}}\left[\lambda_{n}\theta +\lambda_{M}\phi \right]=0,$$

(9) $$\theta^{\prime \prime }+\frac{Pr}{m+1}F\theta^{\prime }+\frac{k_{f}{\left(\rho C_{p}\right)}_{tehnf}}{k_{tehnf}{\left(\rho C_{p}\right)}_{f}}PrEc{\left|F^{\prime \prime }\right|}^{m+1}+\frac{k_{f}{\left(\rho C_{p}\right)}_{tehnf}}{k_{tehnf}{\left(\rho C_{p}\right)}_{f}}PrD_{f}\phi^{\prime \prime }+\frac{k_{f}}{k_{tehnf}}H_{h}Pr\theta =0,$$

(10) $$\phi^{\prime \prime }+\frac{{\left(1-\varphi_{b}\right)}^{-2.5}Sc}{{\left(1-\varphi_{c}\right)}^{2.5}m+1{\left(1-\varphi_{a}\right)}^{2.5}}F\phi^{\prime }-\frac{{\left(1-\varphi_{b}\right)}^{-2.5}Sc}{{\left(1-\varphi_{a}\right)}^{2.5}{\left(1-\varphi_{c}\right)}^{2.5}}K_{c}\phi +ScS_{r}\theta^{\prime \prime }=0.$$

Defined correlations in the motion of tri-hybrid nanoparticles are [34]:

(11) $$\rho_{tehnf}=\left(1-\varphi_{a}\right)\left\{\left(1-\varphi_{b}\right)\left[\left(1-\varphi_{c}\right)\rho_{f}+\varphi_{c}\rho_{3}\right]+\varphi_{b}\rho_{2}\right\}+\varphi_{c}\rho_{1},$$

(12) $$\frac{\mu_{f}}{{\left(1-\varphi_{a}\right)}^{2.5}{\left(1-\varphi_{b}\right)}^{2.5}{\left(1-\varphi_{c}\right)}^{2.5}},\;\frac{K_{tenf}}{K_{nf}}=\frac{K_{2}+2K_{nf}-2\varphi_{a}\left(K_{nf}-K_{2}\right)}{K_{2}+2K_{nf}+\varphi_{b}\left(K_{nf}-K_{2}\right)},$$

(13) $$\frac{K_{tehnf}}{K_{hnf}}=\frac{K_{1}+2K_{hnf}-2\varphi_{a}\left(K_{hnf}-K_{1}\right)}{K_{1}+2K_{hnf}+\varphi_{a}\left(K_{hnf}-K_{1}\right)},\;\frac{K_{nf}}{K_{f}}=\frac{K_{3}+2K_{f}-2\varphi_{c}\left(K_{f}-K_{3}\right)}{K_{3}+2K_{f}+\varphi_{c}\left(K_{f}-K_{3}\right)},$$

(14) $$\frac{\sigma_{tenf}}{\sigma_{hnf}}=\frac{\sigma_{1}\left(1+2\varphi_{a}\right)-\varphi_{hnf}\left(1-2\varphi_{a}\right)}{\sigma_{1}\left(1-\varphi_{a}\right)+\sigma_{hnf}\left(1+\varphi_{a}\right)},\;\frac{\sigma_{hnf}}{\sigma_{nf}}=\frac{\sigma_{2}\left(1+2\varphi_{b}\right)+\varphi_{nf}\left(1-2\varphi_{b}\right)}{\sigma_{2}\left(1-\varphi_{b}\right)+\sigma_{nf}\left(1+\varphi_{b}\right)},$$

(15) $$\frac{\sigma_{nf}}{\sigma_{f}}=\frac{\sigma_{3}\left(1+2\varphi_{c}\right)+\varphi_{f}\left(1-2\varphi_{c}\right)}{\sigma_{3}\left(1-\varphi_{c}\right)+\sigma_{f}\left(1+\varphi_{c}\right)}.$$

(16) $$\rho_{tehnf}=\left(1-\varphi_{a}\right)\left\{\left(1-\varphi_{b}\right)\left[\left(1-\varphi_{c}\right)\rho_{f}+\varphi_{c}\rho_{3}\right]+\varphi_{b}\rho_{2}\right\}+\varphi_{c}\rho_{1},$$

(17) $$\frac{\mu_{f}}{{\left(1-\varphi_{a}\right)}^{2.5}{\left(1-\varphi_{b}\right)}^{2.5}{\left(1-\varphi_{c}\right)}^{2.5}},\;\frac{K_{tenf}}{K_{nf}}=\frac{K_{2}+2K_{nf}-2\varphi_{a}\left(K_{nf}-K_{2}\right)}{K_{2}+2K_{nf}+\varphi_{b}\left(K_{nf}-K_{2}\right)},$$

(18) $$\frac{K_{tehnf}}{K_{hnf}}=\frac{K_{1}+2K_{hnf}-2\varphi_{a}\left(K_{hnf}-K_{1}\right)}{K_{1}+2K_{hnf}+\varphi_{a}\left(K_{hnf}-K_{1}\right)},\;\frac{K_{nf}}{K_{f}}=\frac{K_{3}+2K_{f}-2\varphi_{c}\left(K_{f}-K_{3}\right)}{K_{3}+2K_{f}+\varphi_{c}\left(K_{f}-K_{3}\right)},$$

(19) $$\frac{\sigma_{tenf}}{\sigma_{hnf}}=\frac{\sigma_{1}\left(1+2\varphi_{a}\right)-\varphi_{hnf}\left(1-2\varphi_{a}\right)}{\sigma_{1}\left(1-\varphi_{a}\right)+\sigma_{hnf}\left(1+\varphi_{a}\right)},\;\frac{\sigma_{hnf}}{\sigma_{nf}}=\frac{\sigma_{2}\left(1+2\varphi_{b}\right)+\varphi_{nf}\left(1-2\varphi_{b}\right)}{\sigma_{2}\left(1-\varphi_{b}\right)+\sigma_{nf}\left(1+\varphi_{b}\right)},$$

(20) $$\frac{\sigma_{nf}}{\sigma_{f}}=\frac{\sigma_{3}\left(1+2\varphi_{c}\right)+\varphi_{f}\left(1-2\varphi_{c}\right)}{\sigma_{3}\left(1-\varphi_{c}\right)+\sigma_{f}\left(1+\varphi_{c}\right)}.$$

The surface force at the surface of the wall is:

(21) $$C_{f}=-\frac{\tau_{w}}{U^{2}\rho_{f}},\;\tau_{w}=\left(\frac{\partial u}{\partial y}{\left|\frac{\partial u}{\partial y}\right|}^{m-1}\right),$$

Using the value of $\tau_{w}$ in Equation (21) and:

(22) $$C_{f}=-\frac{{\left(\frac{\partial u}{\partial y}{\left|\frac{\partial u}{\partial y}\right|}^{m-1}\right)}_{y=0}}{U^{2}\rho_{f}},$$

Implementing the value of $\frac{\partial u}{\partial y}$ in Equations (1) and (22) becomes:

(23) $${\left(Re\right)}^{\frac{1}{m+1}}C_{f}=-\frac{{\left(1-\varphi_{b}\right)}^{-2.5}}{{\left(1-\varphi_{a}\right)}^{2.5}{\left(1-\varphi_{c}\right)}^{2.5}}\left[F^{\prime \prime }\left(0\right){\left|F^{\prime \prime }\left(0\right)\right|}^{m-1}\right].$$

Nusselt number (Nu) is modeled as:

(24) $$Nu=\frac{xQ_{w}}{\left(T_{w}-T_{\infty }\right)k_{f}},\;Q_{w}=-K_{Thnf}\left(\frac{\partial T}{\partial y}\right),$$

Using the value of $Q_{w}$, we get

(25) $$Nu=\frac{-xK_{Thnf}{\left(\frac{\partial T}{\partial y}\right)}_{y=0}}{\left(T_{w}-T_{\infty }\right)k_{f}},\;{\left(Re\right)}^{\frac{-1}{m+1}}Nu=-\frac{K_{Thnf}}{k_{f}}\theta^{\prime }\left(0\right).$$

The rate of mass diffusion is:

(26) $$Sc=\frac{xM_{w}}{\left(C_{w}-C_{\infty }\right)D_{f}},\;M_{w}=-D_{Thnf}\left(\frac{\partial C}{\partial y}\right),$$

Now, Equation (26) is reduced as

(27) $$Sc=\frac{-xD_{Thnf}{\left(\frac{\partial C}{\partial y}\right)}_{y=0}}{\left(C_{w}-C_{\infty }\right)D_{f}},$$

(28) $${\left(Re\right)}^{\frac{-1}{m+1}}Sc=-\frac{{\left(1-\phi_{b}\right)}^{-2.5}}{{\left(1-\phi_{a}\right)}^{2.5}{\left(1-\phi_{b}\right)}^{2.5}}\phi^{\prime }\left(0\right).$$

## 3. Numerical Scheme

**Figure 3:**
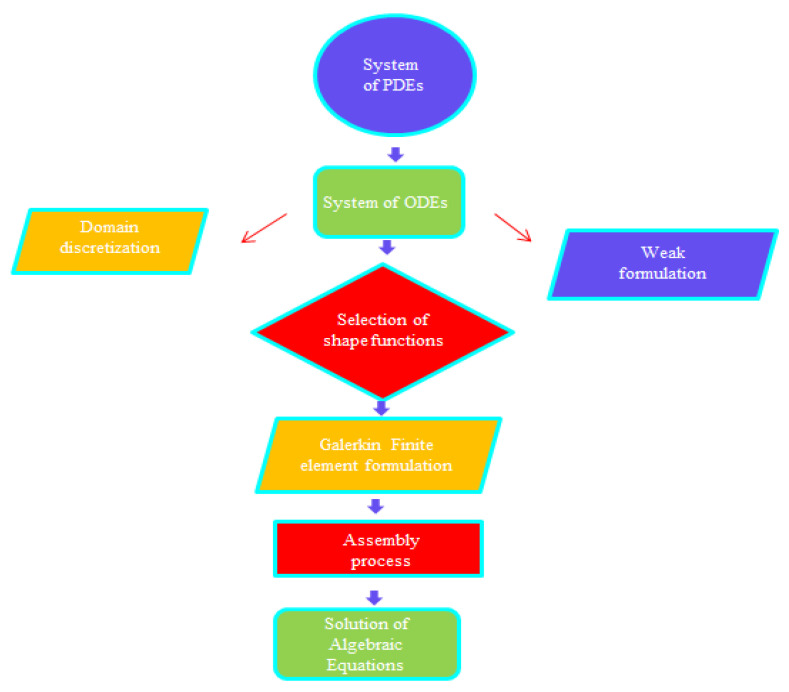
Flow chart regarding the finite element scheme.

The current model’s associated boundary conditions were numerically simulated with the help of a finite element algorithm. The concept upon which the finite element method rests is the division of the required domain into elements (finite). FES (finite element scheme) [8,17] is discussed here. The flow chart of the finite element algorithm is mentioned in Figure 3. This approach has been used in several computational fluid dynamics (CFD) problems; the advantages of using this kind of approach are mentioned below.

⮚ Complex type geometries are easily tackled using the finite element method;
⮚ Physical problems in applied science are numerically solved by finite element formulation (FEM);
⮚ FEM needs a low level of investment in view of time and resources;
⮚ An important role of FEM is to simulate various types of boundary conditions;
⮚ It has the ability to perform discretization regarding derivatives.

### 3.1. Domain Discretization

Firstly, the domain was discretized into small numbers of elements, whereas the approximation solution was made using the concept of the division of elements. This obtained approximation solution is assumed as a linear polynomial.

### 3.2. Choice of Shape Function

The shape function plays a vital role in developing the approximation solution along with the nodal value. The nodal value and the shape function for the solution of the current model are defined as:

(29) $$F=\sum_{j=1}^{ll}\Psi_{n}F_{n},\;\Psi_{n}={\left(-1\right)}^{n-1}\frac{\xi_{n+1}-\xi }{\xi_{n+1}-\xi_{p}},\;\mathrm{here}\;n=1,\;2.$$

### 3.3. Residuals

Observe that the present model is known as a strong form model, whereas weak form models are made via the approach related to GFE (Galerkin finite element). The residual is:

(30) $$\int_{\Omega }\left(\Psi_{a}R\right)d\Omega .$$

### 3.4. Assembly Approach

The concept of an assembly approach is implemented in the development of a global stiffness matrix and stiffness matrices. The linearization of algebraic equations is accomplished via the Picard linearization approach.

### 3.5. Testing of Error Analysis and Mesh Free Analysis

The error analysis of the current investigation is addressed as:

(31) $$Max\left|\Omega_{i}^{r}-\Omega_{i}^{r-1}\right|<10^{-8}.$$

The convergence of problem is ensured within 300 elements. Table 2 captures the convergence of problem.

### 3.6. Validation of Numerical Results

Table 3 depicts the validation of simulations against already published numerical values [35,36,37]. Observe that the present flow model is reduced into flow models [35,36,37] by implementing the values of $\varphi_{a}=\varphi_{b}=\varphi_{c}=0,\;F_{r}=\epsilon =\lambda_{n}=\lambda_{m}=0$ into the current model. So, we have found that there is good agreement between the simulations and previously published works.

## 4. Results and Discussion

The development of the desired model was carried out under the effects of the Soret and Dufour models in a pseudo-plastic material over a vertical frame in the presence of heat energy and mass species transport. A chemical reaction occurred and dual behavior was addressed relating to heat generation and heat absorption. A mixture of silicon dioxide, aluminum oxide, and titanium oxide in ethylene glycol was considered for analysis of the heat energy and mass species characteristics versus the physical parameters. A detailed discussion of the various parameters is illustrated below.

### 4.1. Analysis Related to Motion into Particles

**Figure 4:**
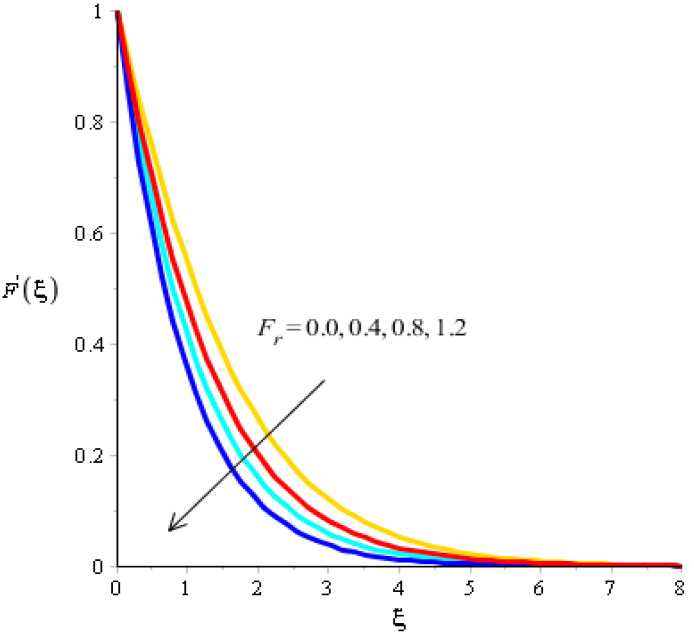
Analysis of velocity curves versus $F_{r}$.

**Figure 5:**
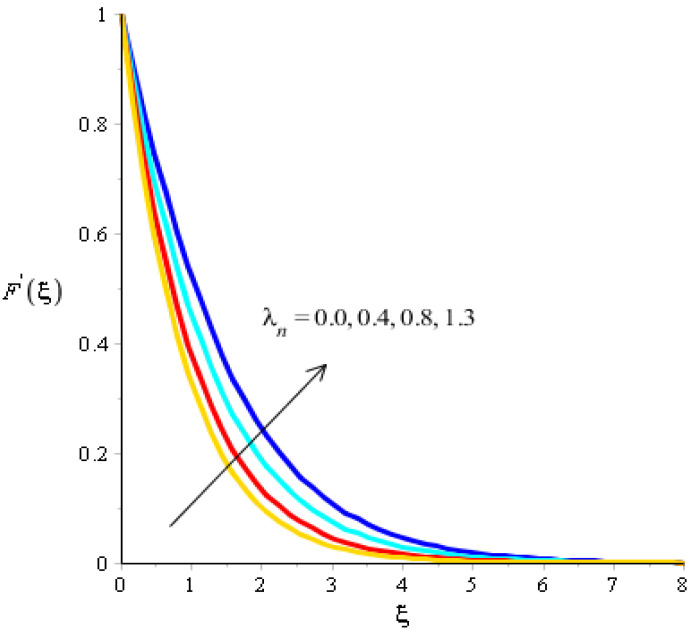
Analysis of velocity curves versus $\lambda_{n}$.

**Figure 6:**
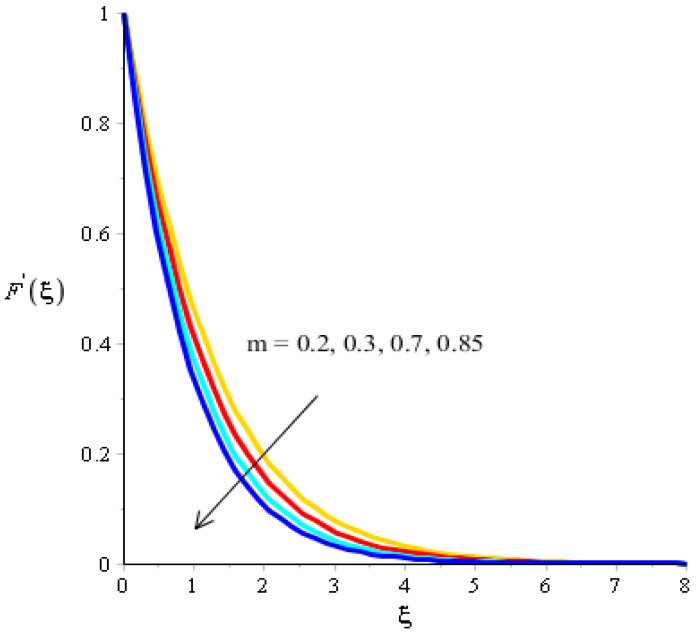
Analysis of velocity curves versus m.

**Figure 7:**
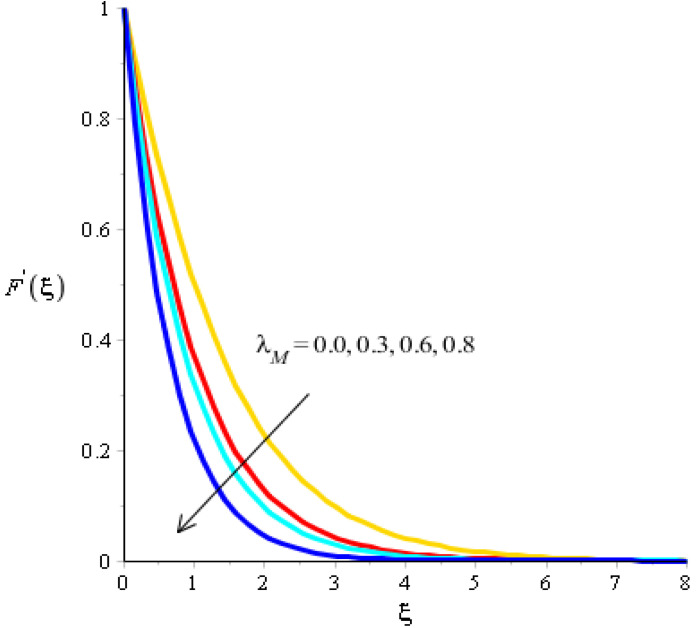
Analysis of velocity curves versus $\lambda_{M}$.

The effect of bouncy force on the flow analysis, an effect of ($F_{r}$) Forchheimer (m) power law, is addressed in Figure 4, Figure 5, Figure 6 and Figure 7. Figure 4 addresses the motion into particles by applying the influence of $F_{r}.$ We observed that the motion of particles became reduced versus the impact of $F_{r}.$ A mixture of silicon dioxide, aluminum oxide, and titanium oxide in ethylene glycol was inserted during the flow of particles. The frictional force became higher when $F_{r}$ was increased. In view of layers, layers regarding momentum at the boundary were decreased versus the impact of $F_{r}.$ Therefore, the fluid became thinner in the case of increasing values for $F_{r}.$ A Forchheimer number was also used for the declination into motion of the fluid particles. It was the most significant in reducing the momentum layers. Physically, it was the ratio of the pressure reduction into fluid particles which was based on inertia and resistance. So, a higher Forchheimer number created a resistance force among the boundary layers of the fluid particles. The role of power law number is visually represented in the motion of particles considered in Figure 6. The behavior of the fluid was based on the values of power law number. It can be observed that the motion was slowed by applying a variation of power law number. Shear thinning, shear thickening, and fluids category were based on values of power law number. For m=0, fluid became Newtonian. So, fluid motion in the case of Newtonian fluid was dominated when compared to non-Newtonian fluid. MBLs associated within momentum were decreased versus the investigation of power law number. The layers became thick versus the impact of power law number. A power law parameter was used significantly to produce a frictional force among the fluid layers. So, frictional force caused a declination in the flow regarding nanoparticles. Further, layers associated with momentum decreased in function against the values of power law number. The effect of buoyancy forces was created due to a vertical heated sheet. The effects related to buoyancy forces on the flow analysis are observed in Figure 5 and Figure 7. Figure 4 details the relation between motion particles and $\lambda_{N}.$ This occurred due to the effect of temperature gradient on the flow analysis. Argumentation related to the motion of particles was boosted when $\lambda_{N}$ was increased. In the case of $\lambda_{N}$, MBLs were also inclined versus an effect of $\lambda_{N}$. An impact of $\lambda_{M}$ was produced due to the effect of a concentration gradient on the flow. However, in this case, the motion regarding particles slowed. A bouncy parameter was generated due to the use of a vertical surface, which was the reason for producing a bouncy parameter. In this case, a gravitational force was placed on the surfaces via perpendicular direction. The direction of the gravitational force and the flow direction were observed as being opposite. Therefore, flow is slowed versus the impact of a bouncy number.

### 4.2. Analysis Related to Thermal Energy into Particles

**Figure 8:**
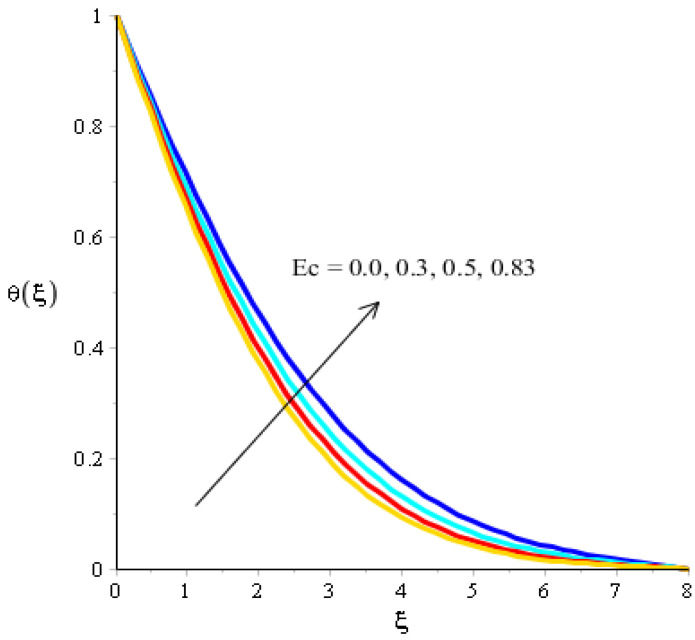
Analysis of the temperature curves versus Ec.

**Figure 9:**
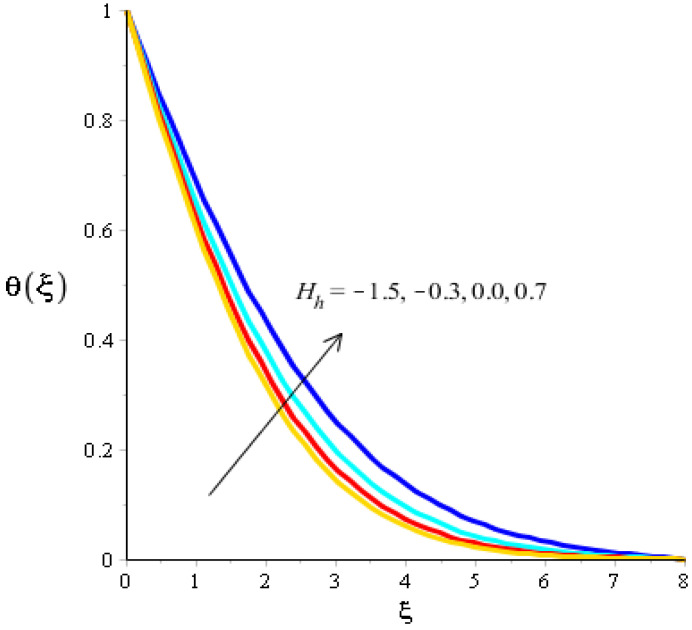
Analysis of the temperature curves versus $H_{h}$.

**Figure 10:**
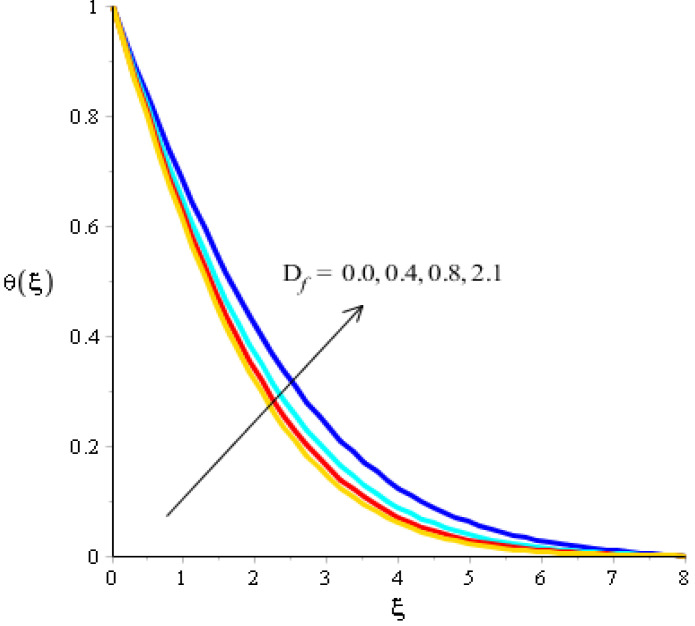
Analysis of the temperature curves versus $D_{f}$.

The effects of Eckert number, the heat generation/heat absorption numbers, and of Dufour number on thermal energy are observed in Figure 8, Figure 9 and Figure 10. A mixture of silicon dioxide, aluminum oxide, and titanium oxide was inserted into ethylene glycol. Figure 8 illustrates the impact of the Eckert number on thermal energy curves inserting ternary hybrid nanoparticles. In this case, heat energy is inclined versus the Eckert number. This inclined impact on temperature curves occurred due to the existence of viscous dissipation. Note that the term regarding viscous dissipation has also been recognized in previously completed work on particles. So, the increase in the viscous dissipation was based on previously completed work. Hence, the work done was increased for the enhancement of particles. Therefore, particles absorb more heat energy when the Eckert number is increased. Thermal layers were based on the viscous dissipation. Higher viscous dissipation created more thermal layers. Hence the increasing function was investigated among the thermal layers and the Eckert number. Figure 9 predicts a dual role regarding heat phenomena. The roles of heat phenomena are known as heat generation and heat absorption, while these behaviors are based on the numerical values of $h_{s}$. Negative numerical values are due to heat absorption, whereas positive numerical values are due to heat generation. Heat energy is boosted when $h_{s}$ is increased because the external source is adjusted at the boundary of the surface. So, due to an external source, heat energy is augmented. The description of the effects of the Dufour number on the thermal layers is illustrated in Figure 10. Heat energy is observed as increasing the function against the variation of the Dufour number. Fluid particles absorb more heat energy in the case of the Dufour number. The comparative investigation between the effects of hybrid nanoparticles, nanostructures, and tri-hybrid nanoparticles on heat energy is considered in Figure 10. A mixture of silicon dioxide, aluminum oxide, and titanium oxide is known as tri-hybrid nanoparticles and a mixture of silicon dioxide and aluminum oxide is known as hybrid nanoparticles, whereas ethylene glycol is the base liquid. In Figure 10, a solid line indicates a tri-hybrid, a dotted line is used for plotting hybrid nanostructures whereas a dashed line is used for nanofluid and a dash-dot line is generated to indicate fluid. We observed that tri-hybrid nanoparticles absorbed maximum heat energy rather than hybrid nanoparticles, fluid, or nanofluid. Hence, tri-hybrid nanoparticles were observed to have more significance for the development and the maximization of heat energy.

### 4.3. Analysis Related to Mass Species

**Figure 11:**
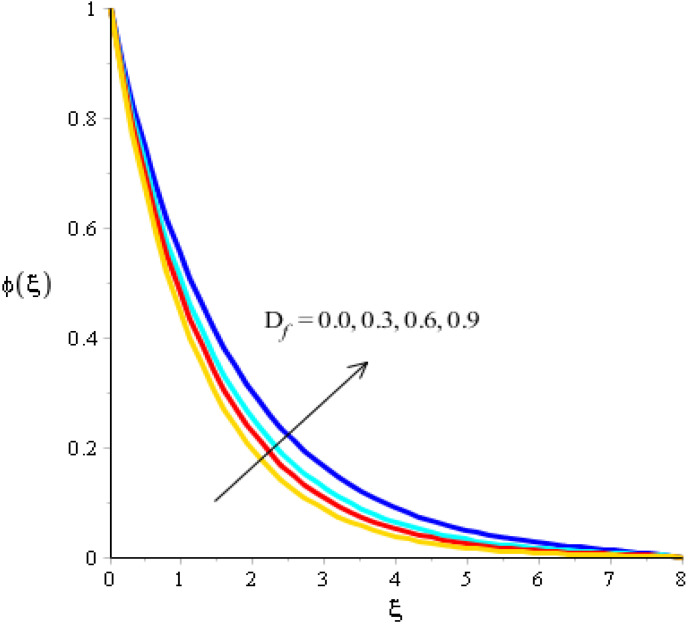
Analysis of concentration curves versus $D_{f}$.

**Figure 12:**
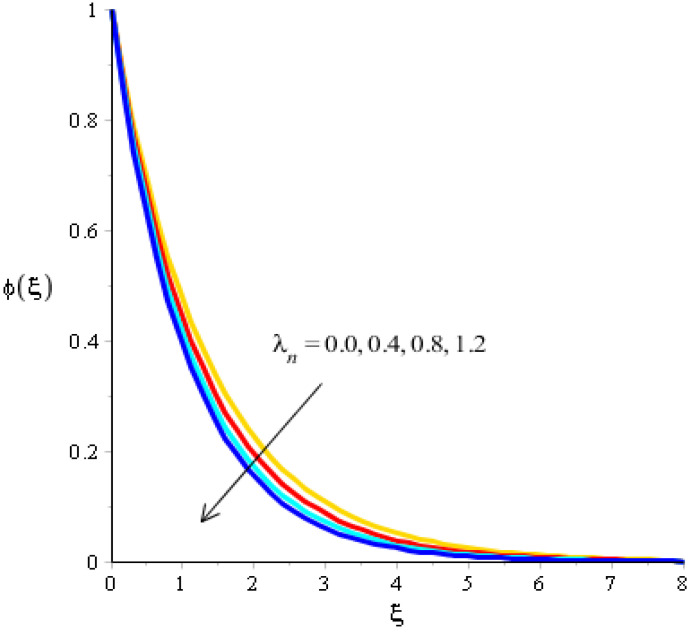
Analysis of concentration curves versus $\lambda_{n}$.

**Figure 13:**
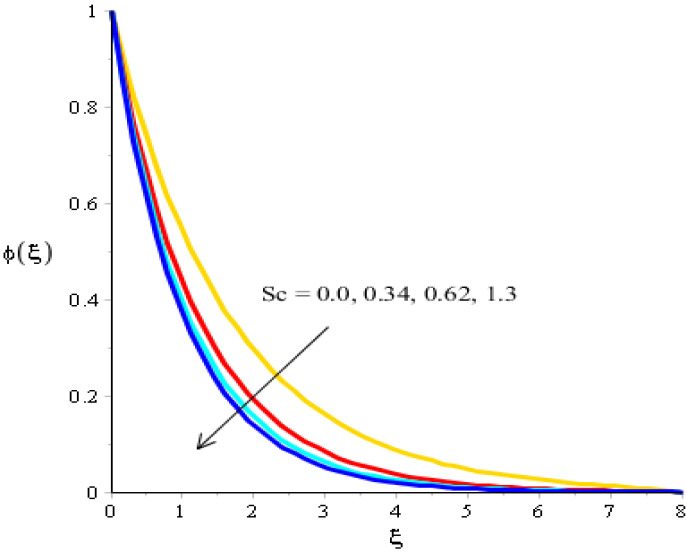
Analysis of concentration curves versus *S_c_*.

**Figure 14:**
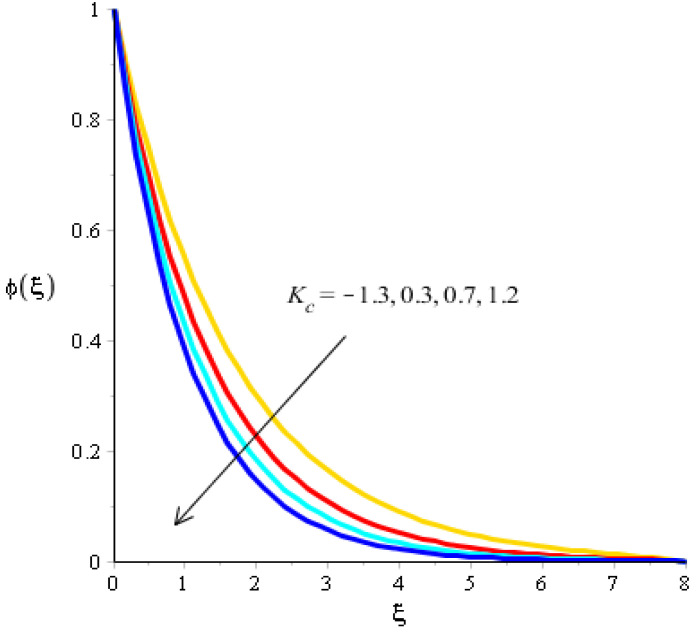
Analysis of concentration curves versus $K_{c}$.

The variations related to the Soret number, Schmidt number, chemical reaction and bouncy force in mass species are captured in Figure 11, Figure 12, Figure 13 and Figure 14. The effect of a Soret number on curves related to the concentration is visualized in Figure 11. Mass species are increased against the distribution in the Soret number. Figure 12 depicts the impact of Bouncy force on mass species. A reduction in mass species versus the distribution in bouncy force was noticed. Figure 13 captures the behavior of the Schmidt number in mass species. The concentration of particles was decreased when Sc was inclined. Physically, Sc is the ratio for mass diffusivity and kinematic viscosity. In view of the physical properties, kinematic viscosity of fluid particles is inclined versus the impact of Sc. However, mass diffusivity is declined against the variation in Sc. Concentration layers have a decreasing function versus the role of Sc. The role of $K_{c}$ on the concentration is estimated in Figure 14, including ternary hybrid nanostructures. The dual character of the chemical reaction on the concentration is observed. Two types of reactions based on destructive and generative reactions were addressed for this case. These reactions were based on the values of $K_{c}$ while the negative values were due to a destructive reaction and the positive values were due to a generative chemical reaction. For both types of reactions, the concentration into particles was declined. Further, the concentration for a destructive reaction was higher than the concentration for a generative reaction.

## 5. Conclusions

A finite element scheme was engaged for handling the modelled physical problem which was past over a stretching sheet containing a mixture of nanoparticles in a pseudo-plastic material under the Soret and Dufour effects. Several important plots were displayed to capture the features of different parameters involved in the fluid velocity, temperature, and concentration fields. Important findings are listed below:

- Confirmation of convergence analysis occurred at 270 elements.
- Forchheimer number, power law number, and $\lambda_{M}$ caused a decline in the thickness of the momentum boundary layer. However, an increment was investigated in the flow versus argument values of the bouncy parameter ($\lambda_{n}).$
- Temperature distribution was maximized versus higher impacts of the Eckert number, heat generation, and the Dufour number while the thickness associated with thermal layers was increased.
- The concentration field was decreased against the argument values of the Schmidt number, chemical reaction number, and bouncy number, whereas the concentration field was enhanced against higher values of the Dufour number.
- The approach of utilizing ternary hybrid nanoparticles was found to be a significant factor in obtaining maximum thermal energy.

## Figures and Tables

**Table 1:** Thermal properties [34] of density, electrical conductivity, and thermal conductivity.

	*K* (Thermal Conductivity)	*σ* (Electrical Conductivity)	*ρ* (Density)
$C_{2}H_{6}O_{2}$	0.253	$4.3\times 10^{-5}$	1113.5
${\mathrm{Al}}_{2}O_{3}$	32.9	$5.96\times 10^{7}$	6310
${\mathrm{TiO}}_{2}$	8.953	$2.4\times 10^{6}$	4250
${\mathrm{SiO}}_{2}$	1.4013	$3.5\times 10^{6}$	2270

**Table 2:** Grid-independent analyses of concentration, temperature, and velocity at mid of 270 elements.

Number of Elements	$F^{\prime }\left(\frac{\xi_{max}}{2}\right)$	$\theta \left(\frac{\xi_{max}}{2}\right)$	$\phi \left(\frac{\xi_{max}}{2}\right)$
30	0.01780117292	0.2638819583	0.1043559742
60	0.02008198265	0.2370957263	0.09636478785
90	0.02028786930	0.2277953868	0.09382548703
120	0.02030867335	0.2231793011	0.09257835690
150	0.02029805160	0.2204312762	0.09183716567
180	0.02028205279	0.2186104527	0.09134595367
210	0.02026649287	0.2173159996	0.09099653209
240	0.02025264615	0.2163487639	0.09073525408
270	0.02024062141	0.2155986751	0.09053255564

**Table 3:** Validation of the numerical results for skin friction coefficient by considering: $\varphi_{a}=\varphi_{b}=\varphi_{c}=0,\;F_{r}=\epsilon =\lambda_{n}=\lambda_{m}=0$.

	Skin Friction Coefficient	Present Work Skin Friction Coefficient
Sakiadis [35]	−0.44375	−0.442735
Fox et al. [36]	−0.4437	−0.443639
Chen [37]	−0.4438	−0.442837

## Data Availability

All supporting data is included in the paper.

## Nomenclature

**Symbols**	**Used for**	**Symbols**	**Used for**
(v,u)	$\mathrm{Velocity}\;\mathrm{components}\;(ms^{-1})$	ν	$\mathrm{Kinematic}\;\mathrm{viscously}\;(m^{2}s^{-1})$
y,x	Space coordinates (m)	T	Temperature (K)
m	Power law number	ρ	$\mathrm{Fluid}\;\mathrm{density}\;(Kgm^{-3})$
g	Gravitational acceleration $\left(ms^{-2}\right)$	K	$\mathrm{Thermal}\;\mathrm{conductivity}\;(Wm^{-1})$
C	$\mathrm{Mass}\;\mathrm{concentration}\;(\mathrm{Kg}m^{-3})$	$C_{\infty }$	$\mathrm{Ambient}\;\mathrm{concentration}\;(\mathrm{Kg}m^{-3})$
$T_{\infty }$	Ambient temperature (K)	μ	$\mathrm{Fluid}\;\mathrm{viscosity}\;(Kgm^{-1}s^{-1})$
Q	$\mathrm{Heat}\;\mathrm{source}\;(JKs^{-1}m^{-3})$	$C_{p}$	$\mathrm{Specific}\;\mathrm{heat}\;\mathrm{capacitance}\;(JKg^{-1}m^{-3})$
D	$\mathrm{Mass}\;\mathrm{diffusion}\;(m^{2}s^{-1})$	BCs	Boundary conditions
$T_{w}$	Wall temperature (K)	$C_{w}$	$\mathrm{Wall}\;\mathrm{concentration}\;(\mathrm{Kg}m^{-3})$
ξ	Independent variable	ODEs	Ordinary differential equations
F	Dimensionless velocity	θ	Dimensionless temperature
ϕ	Dimensionless concentration	φ	Volume fraction
ϵ	Porosity number	$\lambda_{n}$	Bouncy number
$\lambda_{m}$	Bouncy number	Pr	Prandtl number
Ec	Eckert number	$D_{f}$	Dufour number
$H_{h}$	Heat generation number	Sc	Schmidt number
$K_{c}$	Chemical reaction number	$S_{r}$	Soret number
$C_{f}$	Skin friction coefficient	Nu	Nusselt number
tehnf	Tri-hybrid nanoparticles	Re	Reynolds number
CFD	Computational fluid dynamics	FES	Finite element approach
∞	Infinity	σ	$\mathrm{Electrical}\;\mathrm{conductivity}\;(sm^{-1})$
$\varphi_{a},\;\varphi_{b},\;\varphi_{c}$	Volume fractions	R	Residual function
$\Psi_{a}$	Shape function	$Q_{w}$	Heat flux
m	Consistency coefficient	$\tau_{xxy}$	Shear stress
F	Dimensionless velocity field	hnf	Hybrid nanofluid
nf	Nanofluid	tehnf	Tri-hybrid nanoparticles
f	Fluid	a, b, c, 1, 2, 3	Subscripts regarding nanoparticles

## References

[1] Barnes H.A., Hutton J.F., Walters K. (1989). An Introduction to Rheology, Rheology Series 3.

[2] Eberhard U., Seybold H.J., Floriancic M., Bertsch P., Jiménez-Martínez J., Andrade J.S., Holzner M. (2019). Determination of the effective viscosity of non-Newtonian fluids flowing through porous media. Front. Phys..

[3] Rosti M.E., Takagi S. (2021). Shear-thinning and shear-thickening emulsions in shear flows. Phys. Fluids.

[4] Gul T., Shah R.A., Islam S., Ullah M., Khan M.A., Zaman A., Haq Z. (2014). Exact Solution of Two Thin Film Non-Newtonian Immiscible Fluids on a Vertical Belt. J. Basic Appl. Sci. Res..

[5] Hussain F., Hussain A., Nadeem S. (2020). Thermophoresis and Brownian model of pseudo-plastic nanofluid flow over a vertical slender cylinder. Math. Probl. Eng..

[6] Abdelsalam S.I., Sohail M. (2020). Numerical approach of variable thermophysical features of dissipated viscous nanofluid comprising gyrotactic micro-organisms. Pramana.

[7] Sohail M., Naz R. (2020). Modified heat and mass transmission models in the magnetohydrodynamic flow of Sutterby nanofluid in stretching cylinder. Phys. A Stat. Mech. Its Appl..

[8] Chu Y.M., Nazir U., Sohail M., Selim M.M., Lee J.R. (2021). Enhancement in thermal energy and solute particles using hybrid nanoparticles by engaging activation energy and chemical reaction over a parabolic surface via finite element approach. Fractal Fract..

[9] Hina S., Hayat T., Mustafa M., Alsaedi A. (2014). Peristaltic transport of pseudoplastic fluid in a curved channel with wall properties and slip conditions. Int. J. Biomath..

[10] Salahuddin T., Malik M.Y., Hussain A., Bilal S. (2016). Combined effects of variable thermal conductivity and MHD flow on pseudoplastic fluid over a stretching cylinder by using Keller box method. Inf. Sci. Lett..

[11] Alam M.K., Siddiqui A.M., Rahim M.T., Islam S., Avital E.J., Williams J.J.R. (2014). Thin film flow of magnetohydrodynamic (MHD) pseudo-plastic fluid on vertical wall. Appl. Math. Comput..

[12] Hayat T., Iqbal R., Tanveer A., Alsaedi A. (2018). Variable viscosity effect on MHD peristaltic flow of pseudoplastic fluid in a tapered asymmetric channel. J. Mech..

[13] Sohail M., Ali U., Zohra F.T., Al-Kouz W., Chu Y.M., Thounthong P. (2021). Utilization of updated version of heat flux model for the radiative flow of a non-Newtonian material under Joule heating: OHAM application. Open Phys..

[14] Sohail M., Raza R. (2020). Analysis of radiative magneto nano pseudo-plastic material over three dimensional nonlinear stretched surface with passive control of mass flux and chemically responsive species. Multidiscip. Modeling Mater. Struct..

[15] Shafiq A., Khan I., Rasool G., Sherif E.S.M., Sheikh A.H. (2020). Influence of single-and multi-wall carbon nanotubes on magnetohydrodynamic stagnation point nanofluid flow over variable thicker surface with concave and convex effects. Mathematics.

[16] Manjunatha S., Puneeth V., Gireesha B.J., Chamkha A. (2021). Theoretical Study of Convective Heat Transfer in Ternary Nanofluid Flowing past a Stretching Sheet. J. Appl. Comput. Mech..

[17] Nazir U., Sohail M., Hafeez M.B., Krawczuk M. (2021). Significant Production of Thermal Energy in Partially Ionized Hyperbolic Tangent Material Based on Ternary Hybrid Nanomaterials. Energies.

[18] Chen Z., Yan H., Lyu Q., Niu S., Tang C. (2017). Ternary hybrid nanoparticles of reduced graphene oxide/graphene-like MoS2/zirconia as lubricant additives for bismaleimide composites with improved mechanical and tribological properties. Compos. Part A Appl. Sci. Manuf..

[19] Zayan M., Rasheed A.K., John A., Muniandi S., Faris A. (2021). Synthesis and Characterization of Novel Ternary Hybrid Nanoparticles as Thermal Additives in H_2_O. ChemRxiv.

[20] Shafiq A., Mebarek-Oudina F., Sindhu T.N., Abidi A. (2021). A study of dual stratification on stagnation point Walters’ B nanofluid flow via radiative Riga plate: A statistical approach. Eur. Phys. J. Plus.

[21] Swain K., Mebarek-Oudina F., Abo-Dahab S.M. (2021). Influence of MWCNT/Fe_3_O_4_ hybrid nanoparticles on an exponentially porous shrinking sheet with chemical reaction and slip boundary conditions. J. Therm. Anal. Calorim..

[22] Mebarek-Oudina F., Fares R., Aissa A., Lewis R.W., Abu-Hamdeh N.H. (2021). Entropy and convection effect on magnetized hybrid nano-liquid flow inside a trapezoidal cavity with zigzagged wall. Int. Commun. Heat Mass Transf..

[23] Warke A.S., Ramesh K., Mebarek-Oudina F., Abidi A. (2021). Numerical investigation of the stagnation point flow of radiative magnetomicropolar liquid past a heated porous stretching sheet. J. Therm. Anal. Calorim..

[24] Dadheech P.K., Agrawal P., Mebarek-Oudina F., Abu-Hamdeh N.H., Sharma A. (2020). Comparative heat transfer analysis of MoS_2_/C_2_H_6_O_2_ and SiO_2_-MoS_2_/C_2_H_6_O_2_ nanofluids with natural convection and inclined magnetic field. J. Nanofluids.

[25] Marzougui S., Mebarek-Oudina F., Magherbi M., Mchirgui A. (2021). Entropy generation and heat transport of Cu-water nanoliquid in porous lid-driven cavity through magnetic field. Int. J. Numer. Methods Heat Fluid Flow.

[26] Mebarek-Oudina F. (2019). Convective heat transfer of Titania nanofluids of different base fluids in cylindrical annulus with discrete heat source. Heat Transf. Asian Res..

[27] Dhif K., Mebarek-Oudina F., Chouf S., Vaidya H., Chamkha A.J. (2021). Thermal Analysis of the Solar Collector Cum Storage System Using a Hybrid-Nanofluids. J. Nanofluids.

[28] Zamzari F., Mehrez Z., Cafsi A.E., Belghith A., Quéré P.L. (2015). Entropy generation and mixed convection in a horizontal channel with an open cavity. Int. J. Exergy.

[29] Li Y.X., Al-Khaled K., Khan S.U., Sun T.C., Khan M.I., Malik M.Y. (2021). Bio-convective Darcy-Forchheimer periodically accelerated flow of non-Newtonian nanofluid with Cattaneo–Christov and Prandtl effective approach. Case Stud. Therm. Eng..

[30] Mehrez Z., El Cafsi A. (2021). Heat exchange enhancement of ferrofluid flow into rectangular channel in the presence of a magnetic field. Appl. Math. Comput..

[31] Khashi’ie N.S., Arifin N.M., Sheremet M., Pop I. (2021). Shape factor effect of radiative Cu–Al_2_O_3_/H_2_O hybrid nanofluid flow towards an EMHD plate. Case Stud. Therm. Eng..

[32] Esfe M.H., Esfandeh S., Kamyab M.H., Toghraie D. (2021). Analysis of rheological behavior of MWCNT-Al_2_O_3_ (10: 90)/5W50 hybrid non-Newtonian nanofluid with considering viscosity as a three-variable function. J. Mol. Liq..

[33] Mehrez Z., El Cafsi A. (2017). Thermodynamic Analysis of Al_2_O_3_–Water Nanofluid Flow in an Open Cavity Under Pulsating Inlet Condition. Int. J. Appl. Comput. Math..

[34] Algehyne E.A., El-Zahar E.R., Sohail M., Nazir U., AL-bonsrulah H.A.Z., Veeman D., Felemban B.F., Alharbi F.M. (2021). Thermal Improvement in Pseudo-Plastic Material Using Ternary Hybrid Nanoparticles via Non-Fourier’s Law over Porous Heated Surface. Energies.

[35] Sakiadis B.C. (1961). Boundary-layer behavior on continuous solid surfaces: II. The boundary layer on a continuous flat surface. AiChE J..

[36] Fox V.G., Erickson L.E., Fan L.T. (1968). Methods for solving the boundary layer equations for moving continuous flat surfaces with suction and injection. AIChE J..

[37] Chen C.H. (1999). Forced convection over a continuous sheet with suction or injection moving in a flowing fluid. Acta Mech..

